# Crown feature effect evaluation on wind load for evergreen species based on laser scanning and wind tunnel experiments

**DOI:** 10.1038/s41598-022-25960-2

**Published:** 2022-12-12

**Authors:** Can Lai, Bing Xiao, Jialang Feng, Longyuan Wang, Yanjing Zhang, Yanjun Sun, Xiaoxi Chen, Wei Guo

**Affiliations:** 1 School of Horticulture and Landscape Architecture, Zhongkai University of Agriculture and Engineering, Guangzhou, 510225 China; 2 Shenzhen Landscape Co., Ltd., Shenzhen, 518021 Guangdong China

**Keywords:** Urban ecology, Natural hazards

## Abstract

The wind load a tree withstood is mainly applied to its crown, whose morphology and structure directly affect the degree of wind load given a certain wind condition. Though the features of tree crown are relatively easy to measure, however, among them which is/are the determining factor and how they contribute to wind load remain unknown. In order to figure out how crown features of different tree species influence the wind load, the wind tunnel experiment was performed for 7 most used urban greening tree species, and laser scanning was used to measure the accurate crown features. The results derived by multiple linear model showed (1) *Ficus concinna, Dracontomelon duperreanum*, *Ormosia pinnata* and *Bischofia javanica* are recommended in urban greening for suffering the smaller wind load under the same conditions, whereas *Schefflera macrostachya*, *Acacia confusa* and *Khaya senegalensis* are inadequate towards the view of crown features; (2) crown features like crown horizontal ratio, windward side projection and porosity ratio are important in estimating wind load. Our study demonstrated that evaluating the wind load via crown features is feasible, and provided valuable suggestion for selecting idealized decorative trees in urban environment with a smaller wind load due to the crown features.

## Introduction

Typhoon is a kind of destructive tropical cyclone, which often comes with strong storms, even floods and debris flow, and results in huge life and economic loss for urban areas^1^. With climate change, typhoon occurs more frequently and becomes more destructive, leading to increasingly serious loss for human society. Urban greening trees play an important role in the city ecosystem and contribute to a considerable part of carbon sequestration. Trees are often broken, uprooted or get other irreversible damage due to typhoon. As recorded, the super typhoon “Vicente” in 2012 resulted in the damage of 115 thousand trees^2^. Typhoon “Mangkhut” in 2018 damaged 21.4% of the trees in Shenzhen and tree crown biomass was reduced by 8.44%^3^. The fallen trees disrupted traffic and damaged facilities, whose clearing and recovering cost considerable manpower, materials and finances.

To this end, it is expected that urban greening trees with high wind resistance can be selected and widely adapted to reduce the loss caused by typhoon. Many post-typhoon surveys were carried out to select wind resistance species^4–6^. It is found that the species survived in strong winds have some general features such as open crowns, shorter heights, large branching angle, deep roots, larger stem diameter, compact wood density, etc.

Apart from the survey, similar results regarding wind resist characteristics were also reported by a lot of theoretical and experimental studies. Loehle^7^ summarized that crown shape is important in wind reception, in which trees with conical crowns suffered less drag force than those with cylinder crowns. Based on the quantified horizontal crown profile and vertical crown profile, wind load had different variation forms^8^. Though many studies have emphasized the importance of tree crown in evaluating the tree stability, there is a discrepancy in what is the profitable feature. For example, trees with larger crown width were regarded as vulnerable to wind events by Ver Planck and MacFarlane^9^. However, Dunham and Cameron^10^ hold a different idea, and they found trees with larger crown width are less damaged by wind. There are also the controversies on the effects of crown length on wind vulnerability^11,12^. However, as discussed by Päätalo et al.^13^, the comparison of the wind resistance between Norway spruce and Scot pine was embarrassing, as Norway spruce has a pair of contrasted wind resistant features- longer crown length and wider crown width, which implies the evaluation on wind resistance should take factors synthetically rather than individually.

In addition to the crown structural features mentioned above, other crown characteristics might have the impact on the wind load. Wind load is one of the factors resulting the tree damage, which is measured based on bending moment observations on the stem^14^. As Angelou et al.^15^ reported, the presence or lack of leaves in the crown differed the variation rate of wind load with respect to wind speed. The exploring of tree stability is challenging in situ due to the complicated natural environments^16^. Nevertheless, wind tunnel equipment provides the opportunity to simulate wind environments, which is broadly used in studying the wind load on trees. He and Li^17^ found crown features crown porosity ratio and windward side projection area had a significant negative and positive relationship with wind load in shrub *Murraya exotica*, respectively. The importance of crown porosity ratio was also reported by Cao et al.^18^ using the wind tunnel methods, but among three studied shrubby species, different trends of wind load (drag coefficient) versus crown porosity were showed. Other studies also proved that crown ratio (horizontal ratio and vertical ratio) had effects on wind load^19–21^. Thus, wind tunnel test is an effective quantitative method for studying tree stability under different treatments. In general, amount of studies had suggested tree crown features play a crucial role in wind resistance, which influences the wind reception, tree gravity center, and total mass. But among the crown features, which is/are the decisive factor remains unknown.

Tree crown differs significantly among species, and among sites, which is the selective result of the environment. It has long been documented in the function of photosynthetic capacity, biomass accumulation, competitive strategy, as well as in decorative and stability^22–24^. For the coastal (wind induced) area, selecting suitable trees towards the view of crown features is a crucial and effective way to reduce the damage caused by wind. But which among the crown features, which is/are the decisive factor remains unknown.

In this study, we focus on the effects of crown features on wind load suffered by tree. Seven commonly used urban greening species in South China were selected as the material for wind tunnel test modeling the wind load under different wind speeds. Laser scanning technology was applied to derive the accurate values of crown features including crown horizontal ratio, crown vertical ratio, windward side projection area, leaf area ratio and porosity ratio. In this work, we would like to (1) evaluate the wind resistance of these widely used 7 species regarding crown; (2) figure out the contribution to wind load of crown features; (3) form the instructions for urban greening species selection and pruning.

## Material and methods

### Tree samples

Seven evergreen species were selected as the experimental material in this study, including *Bischofia javanica (BJ)*, *Schefflera macrostachya (SM)*, *Acacia confuse (AC)*, *Ormosia pinnata (OP)*, *Ficus concinna (FC)*, *Khaya senegalensis (KS)* and *Dracontomelon duperreanum (DD)*, which are widely used in urban greening in the south of China. The selected 7 tree species have 6 different types of crown shapes including round oval shape, layered-umbrella shape, bell shape, umbrella shape, oval shape and obovate shape, and also cover compound leaf (ternate, palmate and pinnate) and simple leaf (Table 1). Besides, these 7 species differ considerably in the extent of damage caused by the past typhoon. For each species, 3 sample trees were randomly chosen from the area beside the Qianhai stone Park, Baoan District, Shenzhen City. The DBH, tree height, crown length and crown diameter of sample trees were 102.2–167.0 mm, 4.00–7.33 m, 2.12–5.95 m, 2.29–3.77 m, respectively. Since the whole tree is too large for the wind tunnel experiment, tree-like pieces were cut from trees as the reduced-scale experimental materials. This is justified by the fact that trees are typically found to have fractal-like branches^25,26^. The common pattern at different scales in plants had been formalized in quantitative theories proposing a scale-free relationship^27–29^. Furthermore, wind resistance had been demonstrated to determine the self-similar and allometric scaling similar pattern of trees^30^. These indicate that any “entire” fraction of the tree, no matter what Strahler order it is, should follow a similar allometric relationship with the tree, i.e. the fraction can be regarded as a reduced-scale tree-like piece. Tree-like pieces have been considered in other research fields, such as pollutant dispersion^31,32^. The permission was obtained from the administrative office of Qianhai stone Park to collect the plant species, and the use of plants in the present study complies with international, national and/or institutional guidelines.

**Table 1:** Basic information of sample trees.

Species	Crown shape	Leaf type	DBH (mm)	Tree height (m)	Crown length (m)	Crown diameter (m)
*Bischofia javanica*	Round oval shape	Ternate	102.2(2.65)^d^	5.58(1.80)^b^	3.75(2.16)^c^	3.04(0.81)^abc^
*Schefflera macrostachya*	Layered-umberalla shape	Palmate	108.7(1.94)^cd^	6.33(1.40)^ab^	3.73(2.23)^c^	2.29(0.77)^c^
*Acacia confusa*	Umberalla shape	Pinnate	105.2(2.93)^d^	7.33(1.29)^a^	5.95(1.51)^a^	2.42(0.52)^c^
*Ormosia pinnata*	Bell shape	Pinnate	149.7(3.76)^ab^	6.25(0.99)^ab^	4.0(1.10)^bc^	3.21(1.74)^bc^
*Khaya senegalensis*	Obovate shape	Pinnate	124.3(2.61)^bcd^	5.58(0.59)^b^	3.17(0.52)^cd^	3.33(0.58)^ab^
*Ficus concinna*	Obovate shape	Simple leaf	167.0(1.87)^a^	4.00(0.55)^c^	2.12(0.49)^d^	2.29(0.29)^c^
*Dracontomelon duperreanum*	Oval shape	Pinnate	135.0(1.34)^bc^	7.00(1.62)^a^	5.1(0.30)^ab^	3.77(0.51)^a^

Values in parentheses are stand deviations. Means followed by the same letter are not different at *p* = 0.05 (Kruskal–Wallis test). Values sharing the same superscript letters are not significantly different.

The experiment was conducted in May, 2020.

### Crown scan

**Figure 1:**
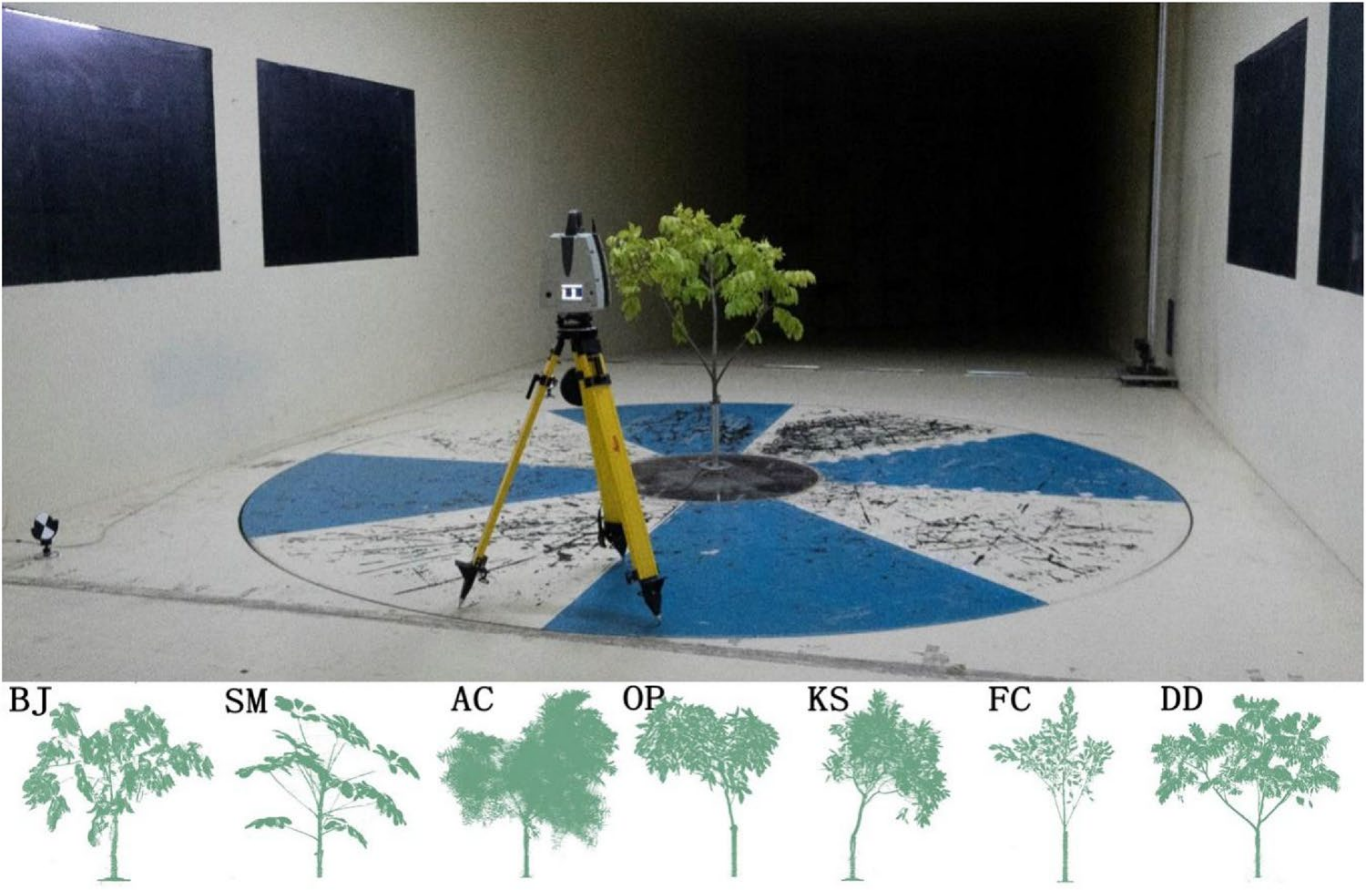
Process of laser scanning and captured photo of 7 species. BJ, SM, AC, OP, KS, FC, and DD are the abbreviations of *Bischofia javanica*, *Schefflera macrostachya*, *Acacia confuse*, *Ormosia pinnata*, *Ficus concinna*, *Khaya senegalensis* and *Dracontomelon duperreanum*, respectively.

A laser scanner (ScanStation P40) was used to acquire the 3D morphology data of trees (Fig. 1). Leica ScanStation P40 terrestrial scanner is with fast scan rate of 1 million points per second at ranges of up to 270 m, 3D position accuracy of 3 mm@50 m (6 mm@100 m), 360° horizontal view and 290° vertical view. The windward side morphology data were imported to Photoshop, and metrics of the windward side of the tree, e.g. the windward projected area, porosity area, leaf area, etc., can be obtained through pixel statistics and equal proportion scaling. The procedures of tree crown scanning are given below:

1. Fix the experimental branches, i.e. the fractal-like piece, on the foundation of wind tunnel laboratory;
2. Set 3 scan markers in the laboratory, take photos for the tree from the front, back, left and right facades, respectively;
3. Put the scanner in front of the tree;
4. Carry out scanner leveling and auto-zeroing, tune the markers to face the scanner;
5. Start scanning until the scanner stops;
6. Move the scanner to the back of the tree, repeat steps (4) and (5);
7. Import the front and back scanning data into Cyclone software and construct the 3D model of the tree based on the markers, the 3D model can be exported as a dxf file;
8. Import the dxf file into AutoCAD software, locate the windward side based on the photos taken in step (2), plot a line of reference and record its actual length (L0), export the windward data and the line of reference as an eps file;
9. Read the eps file using Photoshop, remove the pixels of the steel tube used for fixing and other noise pixels; based on the remaining pixels, create layer 1 where the dense areas are filled, create layer 2 where the empty space is identified by the magic wand tool, create layer 3 where a square with the line of reference as an edge is plotted; then, the areas in three layers are computed using the histogram statistics provided by Photoshop, including the area of leaves and branches (SA) in layer 1, the area of porosity space (SB) in layer 2 and the area of the square (S1) in layer 3; at last, measure the length of the line of reference, L1, using the ruler in Photoshop which is different from L0 due to the rescaling of Photoshop.
10. Covert the measured values into the actual scale and compute the metrics as follows:
  $${\text{Square}}\;{\text{area}}:{\text{S}}0 = {\text{S}}1 \times {\text{L}}0^{2} /{\text{L}}1^{2}$$
  $${\text{Windward}}\;{\text{side}}\;{\text{leaf}}\;{\text{area}}:{\text{SA}}0 = {\text{S}}0 \times {\text{SA}}/{\text{S}}1$$
  $${\text{Windward}}\;{\text{side}}\;{\text{porosity}}\;{\text{area}}:{\text{SB}}0 = {\text{S}}0 \times {\text{SB}}/{\text{S}}1$$
  $${\text{Windward}}\;{\text{side}}\;{\text{projection}}\;{\text{area}}:{\text{SC}} = {\text{SA}}0 + {\text{SB}}0$$
  $${\text{Windward}}\;{\text{side}}\;{\text{porosity}}\;{\text{ratio}}:\rho = \left( {{\text{SB}}0/{\text{SC}}} \right) \times 100\%$$

### Wind tunnel tests

**Figure 2:**
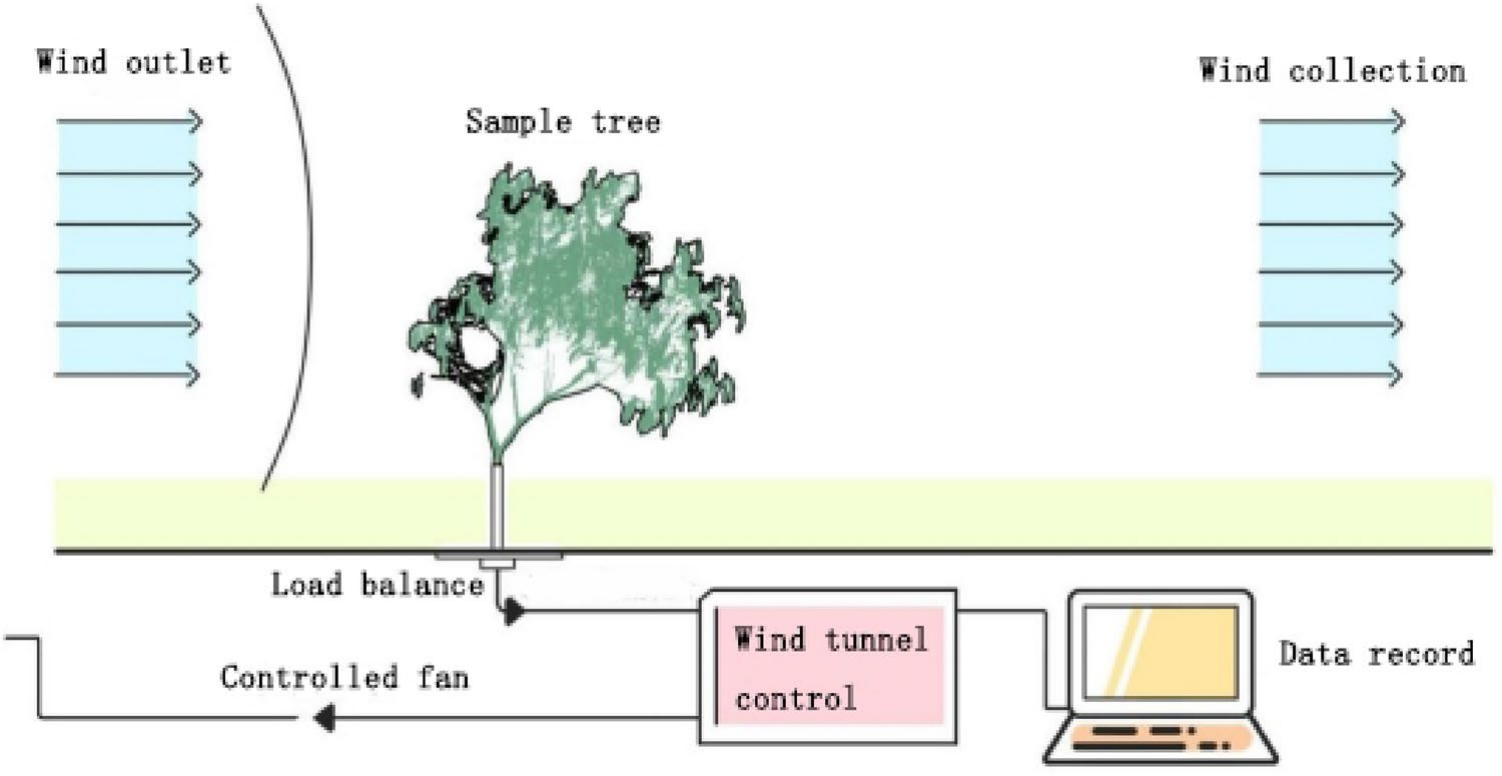
Schematic diagram of wind tunnel test.

The wind tunnel test section has a size of 24 m (L) × 6 m (W) × 3.6 m (H). The wind speed is continuously adjustable within the range of 1.5–30 m/s. The turbulence intensity is less than 1% and the static pressure gradient along tunnel axis is less than 0.01 m^−1^. During the wind tunnel tests, the wind load is measured using a load balance (ATI DELTA) with 8 kHz sampling frequency and ± 0.5% precision. The wind tunnel test was conducted following the procedure below: (1) Cut off three fractal-like branches, which are around 1.5 m tall and like small trees, from each sample tree; (2) Each branch, together with load sensors, was fixed to a base in the wind tunnel laboratory; (3) The wind tunnel test was performed under 5 different wind speeds: 4, 6, 8, 10, 12 m/s, corresponding to light breeze, gentle breeze, moderate breeze, fresh breeze and strong breeze, respectively (referring to Beaufort Scale: level 2 to level 6). The setting of wind speeds in this study is based on the record of wind speed tree suffered in urban green space during typhoon period^33^, and considering the complicated urban wind field in typhoon where wind speed is smaller at a lower height^34^. The 3-axis forces on the piece caused by the wind were measured using load balance. In each experiment, the wind speed was first set to 4 m/s, and the average forces on the piece were recorded after the wind speed was stable. The wind speed was raised step by step until all the option values were reached (Fig. 2).

### Analysis

Crown architectural features were integrated for predicting the wind load including branch length (*L*_*b*_), crown horizontal ratio (*CHR*), crown vertical ratio (*CVR*), in which crown horizontal ratio and crown vertical ratio were calculated as crown width/branch length, and crown length/branch length, respectively. Apart from architectural features, other parameters like windward side projection area (*PA*), leaf area ratio (*LR*), porosity ratio (*PR*), as well as wind speed (*WS*), and species (*SP*) were used to predict the wind load (*WL*). Two types of models were built. The first one (formula (1)) is the general model which is used to estimate the wind load covering all variables mentioned above. And the second one [formula (2)] is used to derive the determinate variables for wind load under different wind speeds. Both of them are multi-linear models.

1 $$WL = a_{1} + \mathop \sum \limits_{i = 1}^{p} b_{i} \times x_{i}$$

2 $$WL_{nm/s } = a_{2} + \mathop \sum \limits_{i = 1}^{q} c_{i} \times x_{i}$$

$a_{1}$, $a_{2}$, $b_{i}$, $c_{i}$ are the coefficients, $p$ is the number of variables, $q$ is the number of variables excluding wind load. And nm/s includes 4 m/s, 6 m/s, 8 m/s, 10 m/s, and 12 m/s.

A stepwise regression model of “both direction” (R package: car) starts with all variables in model (1) and (2). First, the variance inflation factors (VIF) were applied to check the multicollinearity of all independent variables included in the model. Variables will be eliminated for the next step with VIF > 10. For the variables that passed the VIF test, enter the procedure of stepwise regression. Both direction of stepwise regression was applied in the following procedure for the variables passed the VIF test. Akaike information criterion (AIC) was used to determine the variables incorporated in models. Finally, the Shapiro–Wilk test was applied to check the distribution of residuals, in which *p* > 0.05 pass the evaluation. Coefficient of determination (R^2^) was used to judge the goodness of the fit.

Non-parametric analysis, Kruskal–Wallis test, was applied to test the crown features by different species.

All statistical analyses were performed by using the *R* package software (version 3.4.3).

## Results

### Crown feature

The branch features (reduced-scale of crown features) are different among these 7 species (Table 2). The laser scanning result showed the branch length ranges from 1.27 ± 0.18 to 1.59 ± 0.06 m. *B. javanica* and *D. duperreanum* had significantly different branch lengths, while the other 5 species’ branch lengths were not different from each other. *O. pinnata* and *F. concinna* had relatively narrow crowns, *B. javanica*, *O. pinnata* and *F. concinna* had shorter crowns. The crown ratio includes horizontal and vertical ratio. The horizontal ratio varied from 0.53 to 1.00, where 0.53 indicates the crown width is around the half of branch length (*F. concinna*), whereas 1.00 means the crown width is similar to the length (*B. javanica*). Similarly, when the vertical ratio is 0.45 (*F. concinna*), the crown length is less than the half of branch length, when it was 0.94 (*A. confusa*), the crown length is approaching the branch length.

**Table 2:** The statistics of branch information in 7 species.

Species	Branch length (m)	Crown width (m)	Crown length (m)	Crown horizontal ratio	Crown vertical ratio	Windward side projection area (m^2^)	Leaf area ratio (%)	Porosity ratio (%)
*Bischofia javanica*	1.27(0.18)^b^	1.26(0.17)^ab^	0.94(0.13)^cd^	1.00(0.08)^a^	0.74(0.06)^bc^	0.85(0.24)^ab^	67.3(4.17)^b^	32.86(4.16)^b^
*Schefflera macrostachya*	1.57(0.33)^ab^	1.47(0.16)^a^	1.36(0.12)^a^	0.95(0.10)^a^	0.88(0.11)^ab^	0.92(0.14)^ab^	64.26(6.81)^bc^	35.92(6.84)^ab^
*Acacia confusa*	1.31(0.31)^ab^	1.27(0.21)^ab^	1.22(0.25)^ab^	0.99(0.18)^a^	0.94(0.03)^ab^	1.26(0.53)^a^	67.99(2.61)^b^	32.01(2.60)^b^
*Ormosia pinnata*	1.33(0.18)^ab^	0.93(0.16)^cd^	0.84(0.12)^de^	0.70(0.04)^bc^	0.64(0.11)^cd^	0.61(0.16)^bc^	74.24(1.31)^a^	25.98(0.99)^c^
*Khaya senegalensis*	1.52(0.16)^ab^	1.14(0.05)^bc^	1.11(0.01)^abc^	0.75(0.05)^bc^	0.73(0.07)^cd^	0.98(0.05)^a^	75.48(4.11)^a^	24.94(4.33)^c^
*Ficus concinna*	1.37(0.10)^ab^	0.73(0.19)^d^	0.62(0.13)^e^	0.53(0.11)^c^	0.45(0.08)^d^	0.37(0.05)^c^	56.76(4.57)^c^	42.43(4.16)^ab^
*Dracontomelon duperreanum*	1.59(0.06)^a^	1.21(0.30)^ab^	1.07(0.06)^bc^	0.76(0.16)^b^	0.67(0.05)^cd^	0.93(0.29)^ab^	69.69(3.95)^ab^	30.09(4.15)^bc^

Values in parentheses are stand deviations. Means followed by the same letter are not different at *p* = 0.05 (Kruskal–Wallis test). Values sharing the same superscript letters are not significantly different.

The windward side projection area of crown varied from 1.26 to 0.37 m^2^ in 7 species, where *F. concinna* had the smallest crown profile area. *A. confusa* and *K. senegalensis* had generally larger windward side projection areas with the value of 1.26 (0.53) m^2^ and 0.98 (0.05) m^2^, respectively.

*K. senegalensis*, *O. pinnata* and *D. duperreanum* had significantly lower porosity ratio compared with *B. javanica* and *A. confuse*, in which *O. pinnata* had the lowest porosity ratio, only 25.98%. *F. concinna* has the largest porosity ratio 42.43%. And leaf area ratio presents reversed results with respect to porosity ratio.

### Wind load of wind tunnel test

**Figure 3:**
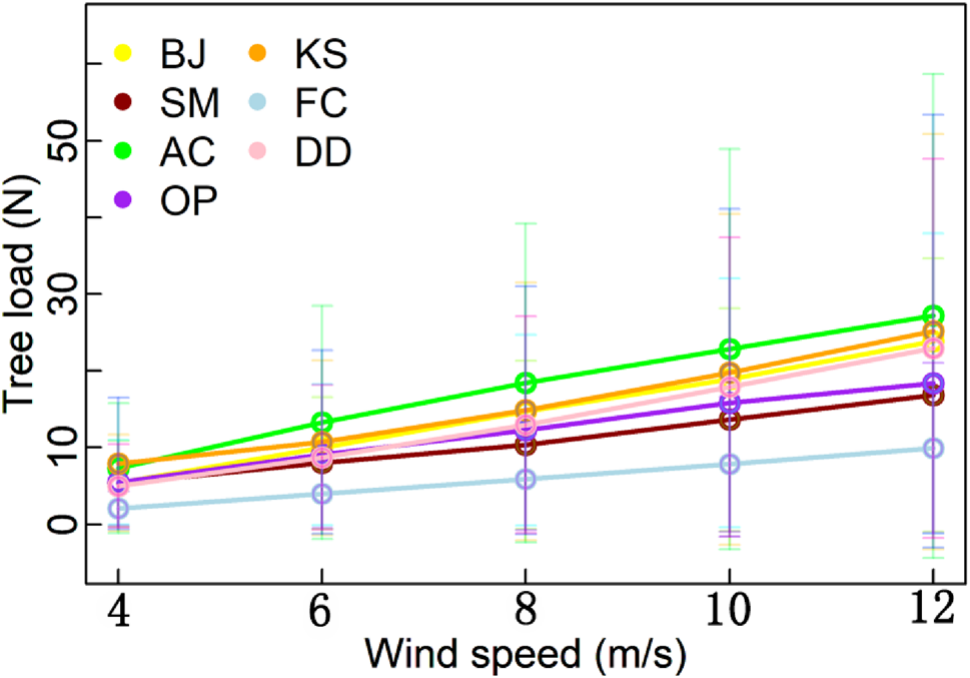
Wind load of different wind speed in 7 species. BJ, SM, AC, OP, KS, FC, and DD are the abbreviations of *Bischofia javanica*, *Schefflera macrostachya*, *Acacia confuse*, *Ormosia pinnata*, *Ficus concinna*, *Khaya senegalensis* and *Dracontomelon duperreanum*, respectively. Wind load showed significant linear correlation with wind speed in 7 species (*p* < 0.01).

During the experiments, there was no breakage and other damages happened. In general, the wind load for all the tree species increased with the wind speed. And wind load showed a significant linear correlation with wind speed in 7 species (*p* < 0.01). *F. concinna* always has the smallest wind load, which gradually increases from 2.03 ± 0.44 to 9.93 ± 2.29 N as the wind speed increases from 4 to 12 m/s (Fig. 3). The wind load of *A. confusa* ranges from 7.32 ± 2.27 to 27.17 ± 8.71 N, which is about three times the wind load of *F. concinna* and is the largest except when the wind speed is 4 m/s. *K. senegalensis* gets the largest wind load 7.92 ± 1.28 N under the 4 m/s speed, but its wind load is less than that of *A. confusa* with respect to larger wind speed. *B. javanica* suffers from wind load almost linearly increasing along with the wind speed. *D. duperreanum* shows similar trend with *B. javanica* but its wind load is smaller. There is an elbow point for the curve of *S. macrostachya* at 8 m/s, after which the increment of wind load becomes larger than before. For *O. pinnata*, the increment of wind load becomes less after 10 m/s.

### Modelling

Among all variables considered in model (1), leaf area ratio and porosity ratio had VIF > 10, indicating their multicollinearity. After removing leaf area ratio, all the rest variables passed the VIF test. Vertical ratio was also excluded due to its failure in significant test. Even though branch length did not pass the significant test (*p* > 0.05), the variable of branch length was suggested to reserve in the step of stepwise regression. Finally, branch length, crown horizontal ratio, windward side projection area, porosity ratio, species, and wind speed were used to construct the model in predicting wind load with R^2^ = 0.873 (Table 3). This model also passed the Shapiro–Wilk test with the *p* = 0.436, indicating the normal distribution of residuals. Thus, the final model is

3 $$WL = 6.24 - 2.73\,L_{b} + 12.18\,PA - 0.23\,PR + 1.89\,WS - 7.13\,CHR - 0.45\,SP$$

**Table 3:** The results of stepwise regression by “both direction” method.

Model	Constant	Branch length	Horizontal ratio	Crown profile area	Porosity ratio	species	Wind speed	R^2^	Adjusted R^2^	F
All	6.24	− 2.73	− 7.13**	12.18**	− 0.23**	− 0.45*	1.89**	0.87	0.87	112.6
4 m/s	10.88**	–	− 4.30*	4.94**	− 0.15**	− 0.35*	–	0.83	0.79	19.93
6 m/s	14.52**	–	− 5.64	7.94**	− 0.16**	− 0.57*	–	0.80	0.75	16.23
8 m/s	22.68**	− 4.76	− 7.40	12.76**	− 0.19*	− 0.47	–	0.82	0.75	13.26
10 m/s	–	–	–	–	–	–	–	–	–	–
12 m/s	18.98**	–	–	15.92**	− 0.37**	–	–	0.81	0.79	38.1

*p* < 0.05 labelled as *; *p* < 0.01 labelled as **.

Several studies also reported that trees with different crown shapes or crown features had different effects on wind load under different wind speeds. Therefore, models under different wind speeds are considered in this study as well. For wind speed 4 m/s and 6 m/s, crown horizontal ratio, windward side projection area, porosity ratio and species all played as important feature in estimating the wind load, and both models turned out to be good fit models with the coefficient of determination 0.83 and 0.80, respectively. However, in 8 m/s, besides the variables mentioned in the model of 4 m/s and 6 m/s, branch length was also included in building the model, but only *α*_2_, windward side projection area and porosity ratio were significant. When wind speed reached 12 m/s, species, and horizontal effects were eliminated but windward side projection area and porosity ratio were still significant in predicting the wind load. Unfortunately, none of the multi-linear model passed the Shapiro–Wilk test of model residuals in wind speed of 10 m/s (*p* < 0.05).

## Discussion

Urban environment differed obviously from natural environment in thermal, water, wind and nutrient conditions for tree growth, which resulted in the difficulties in evaluating wind load for urban greening trees, in addition, the heterogeneity of urban habitats increases the difficulties^35–38^. However, as the exposed part, tree crown can be easily measured compared with belowground parts, and is therefore considered to be practical in wind load estimation^39^. To solve this problem, we designed the above mentioned experiment to explore the correlations of wind load and multi crown features.

Our research incorporated 7 tree species frequently used in South China, covering 6 different crown shapes of round oval, layered-umbrella, bell, umbrella, obovate and oval, as well as the leaf types considering the effect on crown porosity, including ternate, palmate, pinnate and simple leaf (Fig. 1), and showed crown features had the significant effects on wind load. Since the models we constructed by them explained around 80% of wind load (Table 3), all variables included in this study affected the wind load. In general, branch length, horizontal ratio, porosity ratio showed negative effects on wind load. That is to say, when these variables increase, the wind load decreases, and vice versa. In contrast, windward side projection area and wind speed are positively correlated with wind load, indicating as windward side projection area and wind speed increase, the wind load increases consequently. Our results are consistent with other previous studies on tree height, porosity ratio and windward side projection area that shorter heights, larger porosity ratio and smaller windward side projection area are less vulnerable^17,18,20,40^. Turns to crown width, we were not able to provide direct results on how it influences the wind load as the variable we used in this study is horizontal crown ratio instead of crown width considering that crown width varied significantly for different tree heights. Since crown horizontal ratio had a negative relationship with wind load, it indicates trees with larger crown width are favored given the same tree height. Different from other studies, the vertical growth of crown is not as important as the horizontal growth in influencing the wind load in 4–12 m/s as we have shown above.

Apart from crown features adopted in this study, it was reported in many studies^41–43^ that stem diameter also plays a crucial role in determining tree stability, and is a key component in the traditional equation in predicting wind load^35,44^. However, crown width is strongly related with stem diameter, and their relationship is still unclear^45,46^. Furthermore, the stem diameter is highly related with wood density^47,48^, which is not the directly measured variable and showed high variability with other environmental factors like water availability. Thus, stem diameter is not further discussed in the present study.

Consequently, our results implied that in selecting the decorative species in typhoon area for urban planning, trees with the characteristic of short heights, horizontally extended crowns, sparse leaves, and small crown area on windward side are favored. Since crown horizontal ratio is calculated as crown width/branch length, and crown width also influences the windward side projection area, crown width is suggested to be considered integrated with total height. This is also the purpose of current study that evaluates the wind load by multi crown features. Thus, *F. concinna* is an idealized decorative tree with small horizontal ratio (0.53), small profile area (0.37), and high porosity ratio (42.43%) among these 7 selected tree species. Besides, *D. duperreanum*, *O. pinnata* and *B. javanica* also presented wind-resistant crown features. But *S. macrostachya*, *A. confusa* and *K. senegalensis* are not recommended due to the crown features less resisting wind. Besides the species selection in urban planning, our results also provided instructions for tree pruning. Tree pruning is one of the important activities in maintaining the decorative feature as well as the stability^49^. As we mentioned above, in order to enhance the resistance to winds, controlling the total height, reducing the windward side projection area, thinning the branches and leaves inner crowns are profitable. Note that our experimental results provide the pruning instructions merely considering the wind resistance, in practice, the biomechanical design and landscape function should be taken into consideration together^7,50^.

Trees selected in this study showed very different crown architecture and leaf type, thus species effects on estimating wind load are significant in model (1) and models in mild winds, like light breeze, gentle breeze and moderate breeze. Nonetheless, species effect can be ignored when the wind speed reaches in 12 m/s. In such a case, other human interventions like the supporting system should be applied^51^. To be different from other studies, the wind load showed a linear correlation with wind speed in this study rather than allometric or logistic relationship^15,17^. This may because that we only defined the wind speed in the very common range, spanning from light breeze to strong breeze, but not covering all levels of winds. Beyond the linear relationship between crown features and wind load, other relationship observed between trees and wind load are possible and reasonable, as the reasons that result in tree damage are complex. Thus, comprehensive studies incorporating the parameters beside crown features are suggested to carry out in the future for accurate estimations.

## Conclusions

Our study is based on previous studies regarding the effects of several crown features on wind load. The multiple linear model showed that evaluating the wind load by crown features is feasible, and suggested to evaluate the wind load by multi crown features rather than single one. From the perspective of crown features, among the 7 targeted species, *F. concinna* is an idealized decorative tree. Besides, *D. duperreanum*, *O. pinnata* and *B. javanica* also possess the crown features able to suffering smaller wind load. But *S. macrostachya*, *A. confusa* and *K. senegalensis* are not recommended due to the crown features vulnerable to wind. In general, trees with short heights, horizontally extended crowns, sparse leaves, and small windward side projection area are suggested to plant and prune to be used as the decorative trees in urban greening.

Urban is a complicated environment as the habitat for trees. Factors like rooting space, soil properties, water availability, human intervention, etc. have all been attributed to tree growth. Although only considering crown features for vulnerability accessing is far from enough, our results provided valuable information on species selection and pruning activity towards the view of crown features, and this is the relatively easier controllable factor in reducing the damage.

## Supplementary Information

Supplementary Information.

## Data Availability

All data generated or analysed during this study are included in this published article (and its supplementary information files).
